# The C-Terminal Random Coil Region Tunes the Ca^2+^-Binding Affinity of S100A4 through Conformational Activation

**DOI:** 10.1371/journal.pone.0097654

**Published:** 2014-05-15

**Authors:** Annette Duelli, Bence Kiss, Ida Lundholm, Andrea Bodor, Maxim V. Petoukhov, Dmitri I. Svergun, László Nyitray, Gergely Katona

**Affiliations:** 1 Department of Chemistry and Molecular Biology, University of Gothenburg, Gothenburg, Sweden; 2 Department of Biochemistry, Eötvös Loránd University, Budapest, Hungary; 3 Institute of Chemistry, Laboratory of Structural Chemistry and Biology, Eötvös Loránd University, Budapest, Hungary; 4 European Molecular Biology Laboratory (EMBL), Hamburg Outstation c/o DESY, Hamburg, Germany; University of Debrecen, Hungary

## Abstract

S100A4 interacts with many binding partners upon Ca^2+^ activation and is strongly associated with increased metastasis formation. In order to understand the role of the C-terminal random coil for the protein function we examined how small angle X-ray scattering of the wild-type S100A4 and its C-terminal deletion mutant (residues 1–88, Δ13) changes upon Ca^2+^ binding. We found that the scattering intensity of wild-type S100A4 changes substantially in the 0.15–0.25 Å^−1^ q-range whereas a similar change is not visible in the C-terminus deleted mutant. Ensemble optimization SAXS modeling indicates that the entire C-terminus is extended when Ca^2+^ is bound. Pulsed field gradient NMR measurements provide further support as the hydrodynamic radius in the wild-type protein increases upon Ca^2+^ binding while the radius of Δ13 mutant does not change. Molecular dynamics simulations provide a rational explanation of the structural transition: the positively charged C-terminal residues associate with the negatively charged residues of the Ca^2+^-free EF-hands and these interactions loosen up considerably upon Ca^2+^-binding. As a consequence the Δ13 mutant has increased Ca^2+^ affinity and is constantly loaded at Ca^2+^ concentration ranges typically present in cells. The activation of the entire C-terminal random coil may play a role in mediating interaction with selected partner proteins of S100A4.

## Introduction

Through metastasis cancer cells migrate from the primary tumor to distant organs to establish secondary growth and this is considered the most common cause of death amongst cancer patients. The transformation of cancer cells to a metastatic phenotype involves a variety of extracellular and intracellular alterations such as cell signaling, interactions with the extracellular matrix (ECM) and cytoskeleton remodeling. S100A4 (also known as mts1, metastasin, calvasculin, p9Ka) has been strongly associated with increased metastasis properties in several animal models [1], [2].

The interaction of S100A4 to different binding partners (for example p53, annexin A2, CD16, non-muscle myosin II) is mediated by Ca^2+^-induced conformational changes. It has been widely recognized that the conformational change upon Ca^2+^-binding opens up a hydrophobic cleft on the protein surface [3]–[6]. Recently a series of X-ray and a NMR structure of S100A4 complexed with a NMIIA fragment revealed detailed information about the binding sequence and the molecular interactions between S100A4 and the NMIIA fragment [7]–[9].

Mutants with shortened C-terminal regions [10], [11] reduced the ability of S100A4 to bind to NMIIA and to promote metastasis. The deletion of the last two C-terminal amino acids as well as the last fourteen amino acids of S100A4 reduced metastasis formation compared to the wild-type S100A4 [10]. Metastasis was undetectable after the deletion of the 3, 4 and 5 C-terminal amino acids of S100A4 in the same *in vivo* study, while as little as the removal or mutation of the last two residues reduced the binding affinity to NMIIA by an order of magnitude [10]. The crystal and NMR structures determined so far did not provide any clue to rationalize this observation, as the conformation of the C-terminus does not appear to be well defined. As such the C-terminus of S100A4 is one of the most conformationally variable regions in the crystal [3], [8], [12] structures. Through NMR studies of wild-type S100A4 [13], [14] chemical shift differences at the stem of the C-terminal random coil upon Ca^2+^-binding were detected, which was interpreted as an extension of helix 4 by approximately 4 residues (residues 86–90) whereas for the last 11 residues a significant change in chemical shifts was not visible [13], [14]. The relaxation parameters of Ca^2+^-free S100A4 indicated slow dynamics in this region, but this has not been compared to Ca^2+^-bound or target bound states of S100A4. [15].

**Figure 1:**
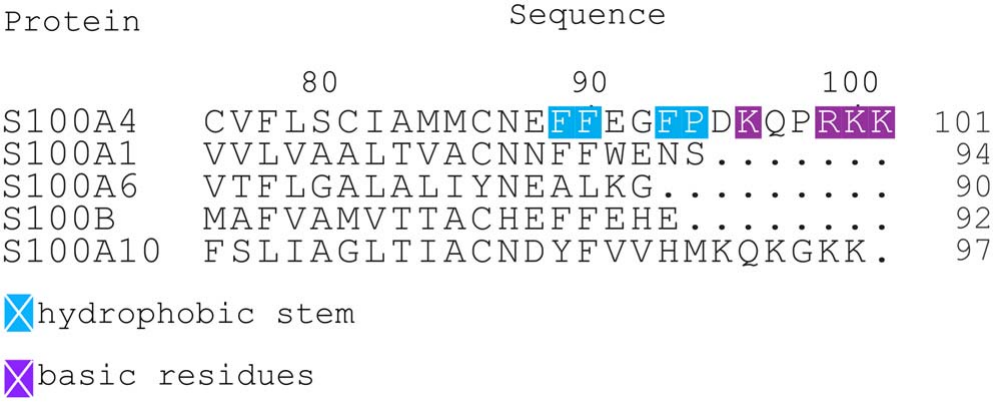
Sequence alignment of several S100 protein family members. The C-terminal region of S100A1, S100A6 and S100B is compared to the last 25 residues of S100A4. The C-terminus of S100A4 consists of a hydrophobic stem and basic tip residues.

The long C-terminal random coil region is characteristic to the S100A4 and S100A10 proteins, the closely related S100A1, S100A6 and S100B proteins have shorter C-terminus. It has a hydrophobic stem (Phe-89, 90 and 93, Pro-94) and a tip populated by bulky basic residues (Lys-96, 100 and 101, Arg-99) (Figure 1). In two crystal structures of Ca^2+^-bound peptide-free S100A4 the hydrophobic cleft of each subunit is occupied by the hydrophobic stem of the C-terminus of an adjacent dimer [6], [12], which may explain the formation of S100A4 oligomers that were observed extracellularly [16]. This cleft is occupied by the NMIIA peptide in the S100A4:NMIIA complex [7], [8] and the hydrophobic stems interact with one other in the crystal forming an elaborate hydrophobic knot between two dimers [8]. Additionally, this unusually long C-terminus with the positively charged tip has a crucial role in annexin A2 (ANXA2) mediated tissue plasminogen activator activation which leads to plasmin formation and finally MMP activation. [5], [17].

Since the role of the C-terminal random coil is not well understood in the mechanism of Ca^2+^- binding we investigated wild-type and truncated S100A4 (Δ13 mutant truncated to residue 88) by solution small-angle X-ray scattering and NMR spectroscopy and rationalized the observed changes by molecular dynamics simulations. We also examined how the deletion of the C-terminal region affects Ca^2+^-binding by isothermal titration calorimetry and solved the crystal structure of Δ13 mutant in complex with the NMIIA interacting peptide.

## Materials and Methods

### Production and Purification of the S100A4 Mutants and the Myosin IIA Fragment

The S100A4 variants and NMIIA fragment were obtained as described in our recently published work [8]. Briefly, the His-tagged human S100A4 variants were expressed in *E. coli* BL21 strain, and purified by Ni^2+^-affinity chromatography. After cleavage of the His_6_-tag with Tobacco Etch Virus protease, the sample were applied to phenyl-Sepharose column, washed thoroughly and eluted with EGTA containing buffer. The purified S100A4 proteins were dialysed against 20 mM Hepes pH 7.5, 20 mM NaCl, 0.1 mM TCEP buffer, concentrated and pooled at −70°C. Prior to SAXS measurements, the S100A4 alone or in complex with MPT peptide were applied to Superdex 75 10/300 GL gel filtration column (GE Healthcare, Little Chalfont, UK) and the peak corresponding to ∼24 kDa molecular weight was collected. The NMIIA fragment MPT (Tyr-Arg1894-Lys1937) was expressed with an N-terminal His_10_-ubiquitin fusion in *E. coli* BL21 strain, and purified by Ni^2+^-affinity chromatography. The His-tagged ubiquitin was removed by Yeast Ubiquitin Hydrolase, while it was dialysed against buffer containing 20 mM TRIS (pH 8.0), 100 mM NaCl and 0.2 mM DTT. The completely digested sample was applied to Ni^2+^-affinity column. The peptide was in the flow-through, which was finally purified by reverse-phase HPLC on a Jupiter 300 C18 column (Phenomenex, Torrance, CA).

### Isothermal Titration Calorimetry

Titrations were carried out in 20 mM Hepes pH 7.5, 150 mM NaCl, and 1 mM TCEP using a Microcal VP-ITC apparatus (GE Healthcare, Little Chalfont, UK). 100 µM S100A4 variants were titrated with 3 mM CaCl_2_ at 310 K. This temperature is optimal to resolve the two binding sites as described previously [6]. 39 injections were performed with 350 sec time intervals between injections. The Origin for ITC 5.0 (OriginLab) software package was used for data processing and the model “Two Set of Sites” was fitted. Peptide binding experiments were carried out at 298 K as described previously [8].

### SAXS Measurement and Data Analysis

SAXS data were collected on the beamlines ID14-3 and BM-29 at the European Synchrotron Radiation Facilities (ESRF, Grenoble, France). Data from protein solutions at a concentration range from 5 mg/ml to ∼1 mg/ml were collected under continuous flow with an X-ray wavelength of 0.931 Å at 283 K and an exposure time of 7 s per frame at ID14-3, and 0.992 Å and an exposure time of 1 s per frame at BM-29. 10 frames for each concentration were recorded (Detector: Pilatus 1 M (Dectris), with 0.01–5 nm^−1^ range). Scattering curves measured at different concentrations were merged and processed in Primus [18]. Theoretical scattering profiles of the high resolution structures were calculated and compared to experimental scattering profiles using the program Crysol [19] with default parameters. Ensembles of S100A4 with constant core domain (residues 1–85) with variable C-terminal region were generated using the program EOM [20]. The calculated intensity from the subset of these ensembles was optimized against the Ca^2+^-free and bound experimental data.

### NMR Measurements and Data Analysis

NMR spectra were collected on a Bruker Avance III spectrometer operating at 700.17 MHz frequency for ^1^H, equipped with a 5-mm ^1^H/^13^C/^15^N probe-head with z-gradient. Typical sample composition was 1 mM protein, 150 mM NaCl, 20 mM Hepes, 2 mM TCEP, 5 mM EGTA, 10% D_2_O. Data were collected on the sample containing the Ca^2+^-free protein, followed by addition of Ca^2+^ solution and the resulting Ca^2+^ concentration was 9 mM. Measurements were done at 287 K, and the temperature was calibrated with standard methanol solution. Diffusion measurements were done with a standard Bruker pulse sequence using stimulated echo, bipolar pulses and 3-9-19 sequence for water suppression. The gradient calibration constant was determined using doped H_2_O sample at 298 K. The sample temperature was stable within 0.01 K during one experiment. 1D ^1^H spectra were recorded in pseudo 2D mode and gradient strength was varied linearly in 32 steps between 2% and 95% of its maximum value. Delays were optimized in order to achieve a total decay in protein signal, thus the applied diffusion time (Δ) was 170 and 200 ms, and gradient length (δ) was 4 and 5 ms. For each sample at least 3 measurements in 8 K data points with 128, 256 or 512 transients were acquired. Data processing and analysis was done in TopSpin. Upon processing only zero order phase correction was applied and integration was performed in regions where no buffer peaks were present. In this respect two chemical shift ranges were chosen: (1.33–1.15) ppm and (0.82–0.20) ppm.

The decay of the protein signal integrated intensity with gradient strength was fitted to a single component. This approach was satisfactory in all cases – meaning homodimers are present in the solution. However, in the case of the Ca^2+^-bound WT protein sample the possible presence of oligomers was checked by fitting two components. Even in this case one component, corresponding to a homodimer, was sufficient to model the data. Diffusion constant calculation was based on fitting the variation of integrated intensity values I as function of gradient strength G_i_ according to the equation:
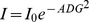
where D is the diffusion constant, A is a constant containing diffusion time, diffusion gradient length values and the calibrated gradient constant. Fitting was done on 29–31 points. From the obtained diffusion constant values, assuming all molecules are spherical, hydrodynamic radii were calculated on the basis of the Stokes-Einstein equation:



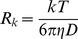
where k is the Boltzmann constant, T the absolute temperature and η is the viscosity of the aqueous solution at the given temperature. For each measurement two diffusion constant values were calculated for the above mentioned two ^1^H chemical shift regions, thus the given values are the average of at least 6 data points. A typical decay curve is shown on Figure S1 and the determined diffusion constants and corresponding R_h_ values are listed in Table S1.

### Molecular Dynamics Simulations

A standard GROMACS protocol was used for construction of the systems in Table S2. Protein molecules were put in a dodecahedron simulation box and solvated in SPC/E water. For all systems, the necessary number of counter ions was added to neutralize system charge and additional sodium and chloride ions were provided until approximately 0.1 M concentration was achieved. The OPLS-AA/L force field was used for all simulations. All systems were energy minimized and subsequently heated up to 300 K temperature. In all simulations a time step of 2 fs was used. The simulations were performed in the isobaric-isothermal ensemble (NPT) and the temperature and pressure (1 bar) was scaled by the modified Berendsen thermostat [21] and the Parrinello-Rahman barostat [22] with 0.1 ps and 2 ps sampling, respectively. The van der Waals and electrostatic interactions were truncated using the Verlet cutoff scheme [23]. The trajectory was updated every 2 ps and each simulation was carried out for 100 ns.

## Results and Discussion

**Figure 2:**
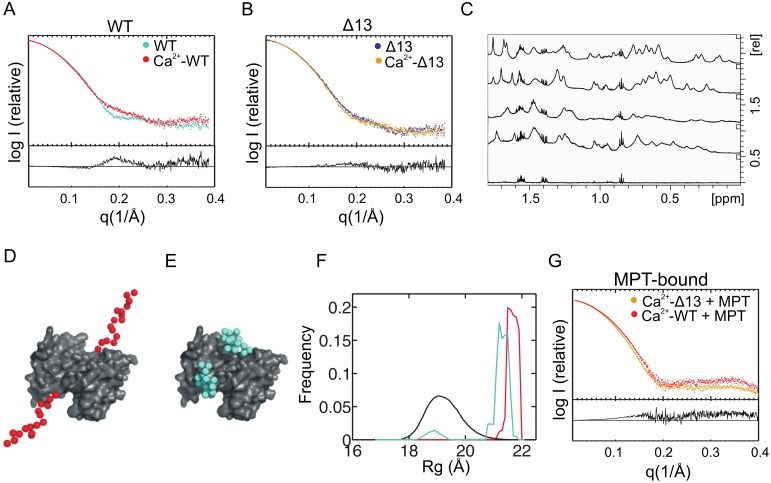
Scattering curves of Ca^2+^-free, Ca^2+^-bound and MPT-bound S100A4. Differences in the WT (*cyan*) and the Ca^2+^-bound WT (*red*) are mainly observed at 0.15 Å^−1^–0.25 Å^−1^ (A). The scattering profile of the Δ13 mutant (*purple*) changes less upon Ca^2+^-binding (*orange*) (B). Typical one-dimensional ^1^H NMR spectra for the studied systems acquired at 700.17 MHz, zoomed to the aliphatic proton region. From bottom to top: buffer; WT; WT-Ca^2+^; Δ13; Δ13-Ca^2+^ (C). Typical examples of EOM [20] extended (D) and compact S100A4 models (E). Distribution of the radii of gyration of the generated model ensemble (*black*) for ensemble optimization method [20], the best ensemble fitting the Ca^2+^-free (*cyan*) and Ca^2+^-bound (*magenta*) WT S100A4 SAXS data (F). Scattering curve differences between the MPT-bound WT (*red*) and the MPT-bound Δ13 mutant (*orange*) are more diffuse (G).

Small angle X-ray scattering (SAXS) and pulsed field gradient NMR spectroscopy were applied in order to study the structural effect of Ca^2+^-binding and the hydrodynamic radii (R_h_) for the S100A4 WT and Δ13 mutant (see Table 1 for notations) in their Ca^2+^-free and Ca^2+^-bound state (Figure 2A, 2B and 2C). While the scattering intensity of the Δ13 mutant did not change substantially upon Ca^2+^-binding (Figure 2B), the wild-type S100A4 SAXS intensity increases noticeably in the 0.15–0.25 Å^−1^ q range. In the protein concentration range (35–113 µM) used in this SAXS experiment the Guinier plot [24] is linear (Figure S2) and the experimental R_g_ values obtained at the lowest concentrations are summarized in Table S1. Modeling an ensemble containing random conformers of the C-terminal region [20] yielded distinct shapes such as shown on Figure 2D and 2E. In the Ca^2+^-bound form an extremely extended conformation of the C-terminal region dominates the modeled ensemble (Figure 2D and 2F) (average R_g_ 22 Å, maximum size 94 Å, χ^2^ 0.81) whereas in the Ca^2+^-free form (Figure 2F) there is a significant peak at the approximate R_g_ of 19 Å representing a more compact conformation (Figure 2E) (average ensemble R_g_ 21 Å, average maximum size 85 Å, χ^2^ 0.89). A similar tendency is shown in the hydrodynamic radii (R_h_) between the Ca^2+^-free (25.6±0.4 Å) and bound form (33.1±1.6 Å) of S100A4, which were calculated from the diffusion coefficients obtained by pulse-field gradient ^1^H-NMR experiments. On the other hand the R_h_ of Δ13 mutant does not change substantially from Ca^2+^-free (25.8±0.3 Å) to Ca^2+^-bound form (24.8±0.6 Å). The aliphatic region of the ^1^H-NMR spectra of the four systems is shown on Figure 2C. The^ 1^H spectra indicate broader peaks for the WT protein compared to the Δ13 mutant. While the addition of Ca^2+^ ions to the Δ13 mutant does not alter the signal intensities, an intensity decrease is observed for the WT protein. This is in line with the formation of oligomers that possess peaks broadening below the detection limit. Data analysis of the diffusion measurements was performed in regions where only protein signals are present (Figure 2C). For the Ca^2+^-free WT and Δ13 mutants the R_h_ values do not differ significantly, indicating both proteins have similar size. This means the longer WT molecule has to be more compact than the shortened mutant. The addition of Ca^2+^ ions causes a small decrease in the R_h_ value for the Δ13 mutant, whereas for the WT protein a considerable increase in size is obtained in the presence of Ca^2+^ in line with a putative extended conformation.

**Table 1:** Description of the proteins and complexes compared in the text and figures.

	Notation	Experimental data	Substitutions	Deletion
**High resolution structures**				
Ca^2+^- and MPT-bound F45WSer	F45WSer	X-ray	C3S, C81S, C86S, F45W	-
Ca^2+^- and MPT-bound Δ13	Δ13Ser	X-ray	C3S, C81S, C86S	F89-K101
Ca^2+^-bound WT	3C1V	X-ray[12]	-	-
Ca^2+^-bound Δ8	4HSZ	X-ray[9]	-	F93-K101
Ca^2+^- and MPT-bound WT	2LNK	NMR[7]	-	- (Ca^2+^ ions not modeled)
Ca^2+^-free WT	1M31	NMR[14]	-	-
**Solution scattering and NMR data**				
WT	WT	SAXS/NMR	-	
Ca^2+^-bound WT	Ca^2+^-WT	SAXS/NMR	-	
Δ13	Δ13	SAXS/NMR	-	F89-K101
Ca^2+^-bound Δ13	Ca^2+^-Δ13	SAXS/NMR	-	F89-K101
Ca^2+^-and MPT-bound WT	Ca^2+^-WT +MPT	SAXS	-	-
Ca^2+^-and MPT-bound Δ13	Ca^2+^-Δ13+MPT	SAXS	-	F89-K101

The relationship between the obtained hydrodynamic radius and radius of gyration for spherical molecules is R_g_ = (3/5)^1/2^ R_h_ [25]. The ratio of 0.77 agrees remarkably well for the Δ13 mutants with R_g_ estimated to be 19.4±0.8 Å and 19.1±1.0 Å for the Ca^2+^-free and Ca^2+^-bound form, respectively based on the Guinier equation. Even a corresponding compression of the R_h_ can be observed upon Ca^2+^ binding (R_h_ of 25.8±0.3 Å and 24.8±0.6 Å, respectively). For the WT protein the agreement is less perfect, indicating that the shape is different. However, the R_g_ of the smaller component from the optimized ensemble of the Ca^2+^-free model (R_g_ approximately 19 Å) is in good agreement with the R_h_ of 25.6±0.4 Å and similar tendencies in size changes obtained from two independent methods illustrating a specific structural role of the C-terminal random coil.

The agreement of the high-resolution structures to the scattering intensities was also evaluated using the program Crysol [19] as described in the Materials and Methods.

The χ^2^ values of the fits of Ca^2+^-free WT solution scattering compared to the Ca^2+^-free WT NMR structure model ensemble (PDB code 1M31) are in the range of 1.85–1.56 (Figure S3A and S3B). For the Ca^2+^-free Δ13 mutant the χ^2^ values are between 1.19 and 1.54, but artificial truncation of the model to compose only residues 1–88, on the other hand, provided a much better fit against the Δ13 mutant data with a χ^2^ range of 0.90–1.09 (Figure S3A and S3C). The Ca^2+^-bound S100A4 SAXS intensity prediction from the crystal structures 3C1V [12], 3CGA [3] and 2Q91 [6] PDB entries are comparable, showing an agreement of a χ^2^ 1.05, 1.19 and 1.04, respectively (Figure S3D).

The SAXS intensity curve of the Δ13 mutant is very similar to the Ca^2+^-bound and free form, which may suggest that either their structure is similar or the limited resolution of the SAXS technique is unable to distinguish clearly these functionally distinct states. On the other hand, if the structures are similar, the Δ13 mutant may be expected to bind MPT with similar affinity in the Ca^2+^-free state as in the Ca^2+^-bound state. Figure S4 and Table S3 show that Ca^2+^-bound Δ13 S100A4, just as the WT protein displays similar affinity to MPT close to the detection limit of the ITC technique, while the Ca^2+^-free state of Δ13 S100A4 does not bind MPT in accordance with the previous findings with WT S100A4 [26]. This finding indicates that the more pronounced SAXS intensity differences between the Ca^2+^-free and bound states of WT S100A4 is related to the differences in the conformational ensemble of the flexible C-terminal random coil. Since the ITC measurements did not reveal the true affinity of MPT to S100A4, we also performed fluorescence polarization measurements. These showed that WT and Δ13 S100A4 have similar affinity to fluorescein labelled MPT with subnanomolar K_d_ of 0.12±0.06 nM (SEM) and 0.28±0.11 nM (SEM), respectively (Figure S5A, B). Similarly, the disassembly of dimeric NMIIA rod filament (fragment 1712Q-1960E) by S100A4 [8] is not affected substantially by the C-terminal truncation (Figure S5C, D).

When a peptide derived from NMIIA (MPT: residues Arg1894-Lys1937) was added to the Ca^2+^-activated WT and the Δ13 mutant the scattering intensity of both variants change similarly (Figure 2G), differences between the wild-type and Δ13 complex are more spread out and difficult to interpret without additional information. Therefore we solved the crystal structure of Ca^2+^-saturated Δ13 mutant in complex with MPT peptide (Supporting information, Figure S6, Table S4), which apart from some differences observed (Supporting information, Figure S7), essentially confirmed the asymmetric binding mode of MPT observed in complexes of the full length protein [7], [8] and Δ8 C-terminal truncated mutant [9].

**Figure 3:**
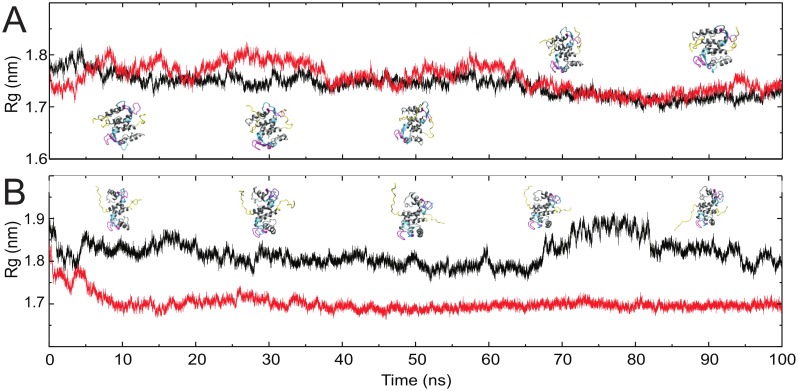
Radius of gyration of 100 ns molecular dynamics trajectories. Ca^2+^-free S100A4 WT (*black*) and AAA constructs (*red*) are compared with snapshots of the structure at 10, 30, 50, 70 and 90 ns the C-terminal random coil, the N- and C-terminal EF-hand are colored *yellow*, *magenta* and *cyan*, respectively (A). Trajectories of the same constructs when in the Ca^2+^-bound form (B).

To shine light on the mechanism of Ca^2+^-activation in the C-terminal random coil molecular dynamics simulations were performed with the GROMACS package [27] on six starting configurations as presented in Table S2. When Ca^2+^ ions were not present the C-termini rapidly became locked on the surface of the core molecule, predominantly associating with the negatively charged residues of the EF hands (Movie S1). As Figure 3 shows similar stabilization was not observed in the Ca^2+^ bound form. Here the C-terminus remained dynamic even after 50 ns. This suggests an electrostatic control of the dynamics of the C-terminal random coil: when Ca^2+^ binds to the EF-hands the Coulombic interaction between the EF-hands and the C-terminus weakens and the C-terminus gains conformational freedom (Movie S2). The trajectories may be biased by the starting configuration; therefore we repeated the simulation with the exact same starting coordinates as before except that the last three residues were replaced by alanines. In this case a curious reversal of the dynamical behavior of the C-terminus occurred: as Figure 3 shows, the Ca^2+^-free trajectory indicates a more dynamic C-terminus whereas in the Ca^2+^-bound form the C-terminus becomes strongly associated by the hydrophobic groove of the Ca^2+^-bound, open form of S100A4. In the absence of positive charges the association with the empty EF hands is broken, but the alanines have a hydrophobic character and a sufficiently small volume to bind into the open S100A4 groove which radically reduces the radius of gyration of the complex and renders it dynamically inactive. This suggests an additional role of the terminal residues: they prevent the autoinhibition by the C-terminus by having a hydrophilic character and their large side chain sterically interferes with the binding. The Δ13 mutant both in the free and Ca^2+^-bound form remained stable for 100 ns in molecular dynamics simulations.

The calculated entropy difference using quasi harmonic approximation between the Ca^2+^-bound and Ca^2+^-free form of S100A4 is −0.60, −1.98 and −1.64 kJ/mol/K, for the WT, AAA and Δ13 constructs, respectively. Even though the diversity of conformations in the immediate vicinity of the Ca^2+^ ions is decreased as compared to the Ca^2+^-free form, and in this respect the C-terminal variants do not differ, in the wild-type C-terminal region the conformational freedom increases upon Ca^2+^-binding leading to a reduced entropy difference between the two states.

**Figure 4:**
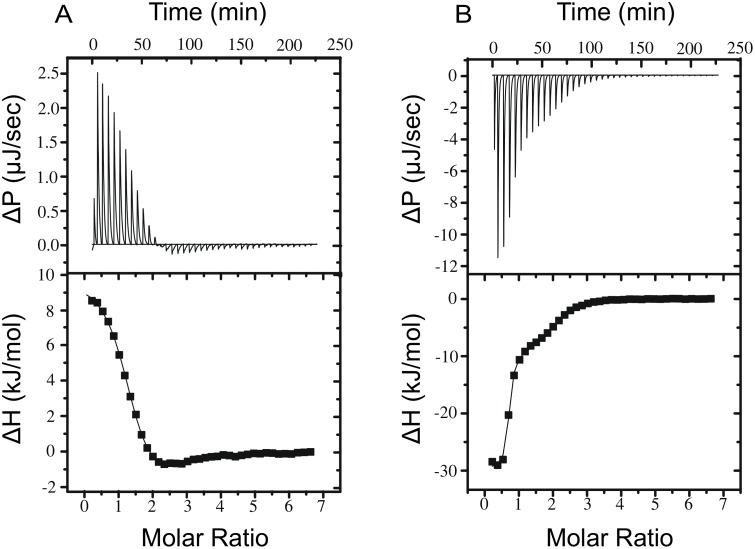
Thermodynamics of Ca^2+^-binding to wild-type (A) and Δ13 (B) S100A4 measured by isothermal titration calorimetry. Plots represent the raw heat effects as a function of time and the binding isotherms as a function of the molar ratio of Ca^2+^ to the protein (100 µM). Calculated thermodynamic parameters are shown in Table 2.

If the S100A4 C-terminus interacts specifically with the EF-hands, it should influence the Ca^2+^-binding properties of S100A4. This is indeed the case: in the Δ13 mutant the Ca^2+^-binding of the high-affinity site is exothermic (in contrast to endothermic Ca^2+^ binding in the WT protein) and the entropic component of the reaction is about one fourth of the value obtained for the wild-type protein (Figure 4A, B) (Table 2). The Ca^2+^-binding property of the wild-type S100A4 is similar to previous observations [6]. This relatively high entropic component of Ca^2+^-binding could be explained by the detachment of the disordered C-terminus. Importantly, the Ca^2+^-binding affinity of the high affinity site is forty times higher in the Δ13 mutant than the affinity determined for the wild-type S100A4. Since the solvent effectively screens long range electrostatic interactions, the removal of positive charges upon the C-terminal truncation alone does not explain the increased affinity. The unstructured C-terminus of S100A4 has to specifically interact with the high-affinity EF hand of S100A4 to reduce the Ca^2+^-affinity and interfere with Ca^2+^-binding. The last 11 residues of C-terminal random coil did not have a specific conformation in the Ca^2+^-free state and switching between EF-hands occurred in the 100 ns simulation, illustrating why it may be difficult to use NMR chemical shifts to model Ca^2+^-dependent local structural changes this region. [13], [14].

**Table 2:** Thermodynamic parameters of Ca^2+^- binding of the S100A4 variants determined by ITC measurements.

	K_d1_	ΔH_1_	n_1_	−TΔS_1_	K_d2_	ΔH_2_	n_2_	−TΔS_2_
	(µM)	(kJ mol^−1^)		(kJ mol^−1^)	(µM)	(kJ mol^−1^)		(kJ mol^−1^)
S100A4+ Ca^2+^	2.7±0.8	10.1±0.1	1.28±0.02	−43.2	30.9±5.7	−4.8±1.2	1.12±0.19	−22.0
S100A4Δ13+ Ca^2+^	0.07±0.01	−29.8±0.3	0.64±0.01	−12.8	11.7±1.8	−10.8±0.5	1.32±0.03	−18.5

The Table displays standard errors of the fits (SEM).

With this high affinity, the C-terminally truncated S100A4 would remain constitutively active inside of the cells and this alone would impair its Ca^2+^-dependent regulatory functions. This may be of more broad ranging significance than putative binding affinity reductions [10] to individual interaction partners such as NMIIA and explain the strong influence of C-terminal truncation of S100A4 in *in vivo* models. This is especially relevant since simplified models of NMIIA such as the shorter NMIIA derived peptide (1908–1923) [9], the MPT peptide in this study did not show significant reduction in binding affinity to truncated forms of S100A4 (Δ8 and Δ13 mutants, respectively). The Δ13 mutant also disassembled filaments of NMIIA fragment 1712Q-1960E with similar efficiency as the wild-type protein. Nevertheless the dynamic Ca^2+^-activated elongated random-coil at C-terminus of S100A4 may be important for interacting with native NMIIA filaments. Binding to other partner proteins such as annexin A2 or p53 may also depend on an intact activated C-terminus in promoting metastasis in tumor cells, which require further studies [5].

The present results provide an insight to the dynamic mechanism of C-terminal random coil in S100A4 as a mediator of S100A4-driven metastasis and shine light on its role in tuning the Ca^2+^-binding affinity of S100A4. These results also suggest that locking the C-terminus to the core domain may be an alternative strategy of inhibiting the metastasis-promoting activities of S100A4.

## Supporting Information

Figure S1 **Typical ^1^H protein signal decay curve as function of the applied gradient strength.** Points indicate measured values, while the continuous line is the fitted curve.
(TIF)

Figure S2 **Guinier plots of the datasets that were merged for further data analysis and modeling.** The Guinier region was extrapolated to the beam stop (*red line*). The data sets are plotted in rings with different colors representing different protein concentrations.
(TIF)

Figure S3 **Comparison of the theoretical SAXS intensities of the high-resolution structures with the experimental SAXS scattering.** The experimental scattering curves are shown in black circles. The theoretical scattering curves were calculated with the program Crysol (21) and are shown in colored lines. The χ^2^-values of the individual models (model number 1–20) of the Ca^2+^-free NMR structure ensemble (PDB code 1M31) [7] were compared to the Ca^2+^-free SAXS scattering curves (A). The calculated scattering curve of the lowest energy NMR model 1M31 against the Ca^2+^-WT SAXS (B) and the Ca^2+^-free Δ13 data (C), respectively. Truncated NMR models are indicated with T. Three Ca^2+^-bound crystal structures (PDB code 3C1V, 2Q91, 3CGA) are also compared to the Ca^2+^-bound WT SAXS scattering curve (D).
(TIF)

Figure S4 **Thermodynamic analysis of S100A4Δ13– MPT interaction.** 75 µM S100A4Δ13 titrated with MPT at 25°C in the presence of 1 mM CaCl_2_ (A) or 1 mM EGTA (B). In the absence of Ca^2+^ no interaction was detected. Calculated thermodynamic parameters are shown in Table S3.
(TIF)

Figure S5 **Fluorescence polarization measurements using (A) WT S100A4 and (B) Δ13 S100A4 and fluorescein labelled MPT peptide.** Optical density changes at 320 nm when NMIIA 1712Q-1960E rod fragment is titrated with (C) WT and (D) Δ13 S100A4.
(TIF)

Figure S6 **Three-dimensional structure of the Ca^2+^-activated, MPT-bound Δ13Ser (A) and F45WSer (B) S100A4.** Subunit A is shown in *green*, subunit B in *blue* and the bound MPT in *yellow*. Helices (H) and the N- and C-terminus (N, C) of the bound peptide are indicated.
(TIF)

Figure S7 **Distance differences in the MPT-bound F45WSer and Δ13Ser.** The superposition of the individual chains was performed with LSQMAN [8]. Distance plot of subunit A and B of S100A4 (A) and the bound MPT peptide (B). The superposition of the three dimensional structure of chain A and chain B are shown in (C) and (D), of the bound peptide is shown in (E).
(TIF)

Table S1 **Experimental R_g_ values and hydrodynamic radius obtained by SAXS and NMR respectively.**
(DOCX)

Table S2 **Specification of the constructs used in different MD simulations.**
(DOCX)

Table S3 **Thermodynamic parameters of peptide binding of the S100A4 variants determined by ITC measurements.**
(DOCX)

Table S4 **Crystallographic table.**
(DOCX)

Movie S1 **MD trajectories of the Ca^2+^-free S100A4**. The time interval visualized is between 20 ns and 100 ns. The last 17 residues of the C-terminal region are shown in red and the EF-hand motifs in cyan.
(WMV)

Movie S2 **MD trajectories of the Ca^2+^-bound S100A4.** The time interval visualized is between 20 ns and 100 ns. The last 17 residues of the C-terminal region are shown in red and the EF-hand motifs in cyan.
(WMV)

Text S1 **Supporting Online Information and Supplementary Methods.**
(DOCX)

## References

[1] Boye K, Maelandsmo GM (2010) S100A4 and metastasis: a small actor playing many roles. Am J Pathol 176: 528–535. 20019188 10.2353/ajpath.2010.090526 PMC2808059

[2] Mishra SK, Siddique HR, Saleem M (2012) S100A4 calcium-binding protein is key player in tumor progression and metastasis: preclinical and clinical evidence. Cancer Metastasis Rev 31: 163–172. 22109080 10.1007/s10555-011-9338-4

[3] Pathuri P, Vogeley L, Luecke H (2008) Crystal structure of metastasis-associated protein S100A4 in the active calcium-bound form. J Mol Biol 383: 62–77. 18783790 10.1016/j.jmb.2008.04.076 PMC2644285

[4] Grigorian M, Andresen S, Tulchinsky E, Kriajevska M, Carlberg C, et al (2001) Tumor suppressor p53 protein is a new target for the metastasis-associated Mts1/S100A4 protein: functional consequences of their interaction. J Biol Chem 276: 22699–22708. 11278647 10.1074/jbc.M010231200

[5] Semov A, Moreno MJ, Onichtchenko A, Abulrob A, Ball M, et al (2005) Metastasis-associated protein S100A4 induces angiogenesis through interaction with Annexin II and accelerated plasmin formation. J Biol Chem 280: 20833–20841. 15788416 10.1074/jbc.M412653200

[6] Malashkevich VN, Varney KM, Garrett SC, Wilder PT, Knight D, et al (2008) Structure of Ca2+-bound S100A4 and its interaction with peptides derived from nonmuscle myosin-IIA. Biochemistry 47: 5111–5126. 18410126 10.1021/bi702537s PMC2633413

[7] Elliott PR, Irvine AF, Jung HS, Tozawa K, Pastok MW, et al (2012) Asymmetric mode of Ca(2)(+)-S100A4 interaction with nonmuscle myosin IIA generates nanomolar affinity required for filament remodeling. Structure 20: 654–666. 22483112 10.1016/j.str.2012.02.002 PMC3343272

[8] Kiss B, Duelli A, Radnai L, Kekesi KA, Katona G, et al (2012) Crystal structure of the S100A4-nonmuscle myosin IIA tail fragment complex reveals an asymmetric target binding mechanism. Proc Natl Acad Sci U S A 109: 6048–6053. 22460785 10.1073/pnas.1114732109 PMC3341023

[9] Ramagopal UA, Dulyaninova NG, Varney KM, Wilder PT, Nallamsetty S, et al (2013) Structure of the S100A4/myosin-IIA complex. BMC Struct Biol 13: 31. 24252706 10.1186/1472-6807-13-31 PMC3924328

[10] Ismail TM, Fernig DG, Rudland PS, Terry CJ, Wang G, et al (2008) The basic C-terminal amino acids of calcium-binding protein S100A4 promote metastasis. Carcinogenesis 29: 2259–2266. 18784356 10.1093/carcin/bgn217

[11] Zhang S, Wang GZ, Liu D, Bao ZZ, Fernig DG, et al (2005) The C-terminal region of S100A4 is important for its metastasis-inducing properties. Oncogene 24: 4401–4411. 15856021 10.1038/sj.onc.1208663

[12] Gingras AR, Basran J, Prescott A, Kriajevska M, Bagshaw CR, et al (2008) Crystal structure of the Ca(2+)-form and Ca(2+)-binding kinetics of metastasis-associated protein, S100A4. FEBS Lett 582: 1651–1656. 18435928 10.1016/j.febslet.2008.04.017

[13] Dutta K, Cox CJ, Huang H, Basavappa R, Pascal SM (2002) Calcium coordination studies of the metastatic Mts1 protein. Biochemistry 41: 4239–4245. 11914069 10.1021/bi012061v

[14] Vallely KM, Rustandi RR, Ellis KC, Varlamova O, Bresnick AR, et al (2002) Solution structure of human Mts1 (S100A4) as determined by NMR spectroscopy. Biochemistry 41: 12670–12680. 12379109 10.1021/bi020365r

[15] Dutta K, Cox CJ, Basavappa R, Pascal SM (2008) 15N relaxation studies of Apo-Mts1: a dynamic S100 protein. Biochemistry 47: 7637–7647. 18627127 10.1021/bi8005048

[16] Novitskaya V, Grigorian M, Kriajevska M, Tarabykina S, Bronstein I, et al (2000) Oligomeric forms of the metastasis-related Mts1 (S100A4) protein stimulate neuronal differentiation in cultures of rat hippocampal neurons. J Biol Chem 275: 41278–41286. 11018041 10.1074/jbc.M007058200

[17] MacLeod TJ, Kwon M, Filipenko NR, Waisman DM (2003) Phospholipid-associated annexin A2-S100A10 heterotetramer and its subunits: characterization of the interaction with tissue plasminogen activator, plasminogen, and plasmin. J Biol Chem 278: 25577–25584. 12730231 10.1074/jbc.M301017200

[18] Konarev PV, Volkov VV, Sokolova AV, Koch MHJ, Svergun DI (2003) PRIMUS: a Windows PC-based system for small-angle scattering data analysis. Journal of Applied Crystallography 36: 1277–1282.

[19] Svergun D, Barberato C, Koch MHJ (1995) CRYSOL - A program to evaluate x-ray solution scattering of biological macromolecules from atomic coordinates. Journal of Applied Crystallography 28: 768–773.

[20] Bernado P, Mylonas E, Petoukhov MV, Blackledge M, Svergun DI (2007) Structural characterization of flexible proteins using small-angle X-ray scattering. J Am Chem Soc 129: 5656–5664. 17411046 10.1021/ja069124n

[21] Berendsen HJC, Postma JPM, Vangunsteren WF, Dinola A, Haak JR (1984) Molecular-Dynamics with Coupling to an External Bath. Journal of Chemical Physics 81: 3684–3690.

[22] Parrinello M, Rahman A (1981) Polymorphic Transitions in Single-Crystals - a New Molecular-Dynamics Method. Journal of Applied Physics 52: 7182–7190.

[23] Verlet L (1967) Computer Experiments on Classical Fluids.I. Thermodynamical Properties of Lennard-Jones Molecules. Physical Review 159: 98–&.

[24] Guinier A (1938) The diffusion of X-rays under the extremely weak angles applied to the study of fine particles and colloidal suspension. Comptes Rendus Hebdomadaires Des Seances De L Acad Des Sci 206: 1374–1376.

[25] Linegar KL, Adeniran AE, Kostko AF, Anisimov MA (2010) Hydrodynamic radius of polyethylene glycol in solution obtained by dynamic light scattering. Colloid Journal 72: 279–281.

[26] Ford HL, Silver DL, Kachar B, Sellers JR, Zain SB (1997) Effect of Mts1 on the structure and activity of nonmuscle myosin II. Biochemistry 36: 16321–16327. 9405067 10.1021/bi971182l

[27] Pronk S, Pall S, Schulz R, Larsson P, Bjelkmar P, et al (2013) GROMACS 4.5: a high-throughput and highly parallel open source molecular simulation toolkit. Bioinformatics 29: 845–854. 23407358 10.1093/bioinformatics/btt055 PMC3605599

