# Supplementary figures and images for: The C-Terminal Random Coil Region Tunes the Ca2+-Binding Affinity of S100A4 through Conformational Activation
Source: PLoS One. 2014 May 15;9(5):e97654. doi: 10.1371/journal.pone.0097654 (PMC4022583; doi:10.1371/journal.pone.0097654)

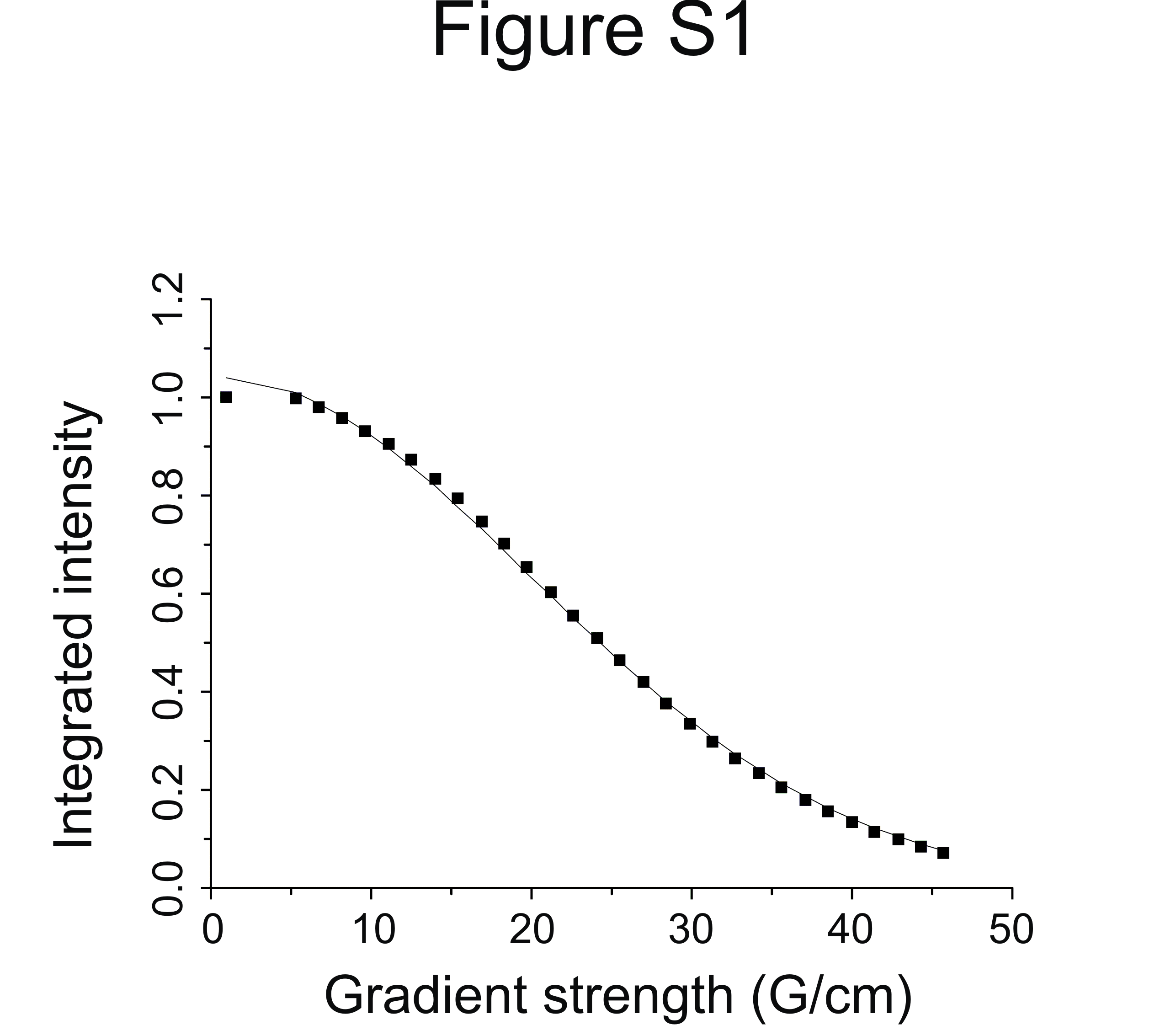

Supplement: Figure S1 — Typical 1H protein signal decay curve as function of the applied gradient strength. Points indicate measured values, while the continuous line is the fitted curve. (TIF) [file pone.0097654.s001.tif]

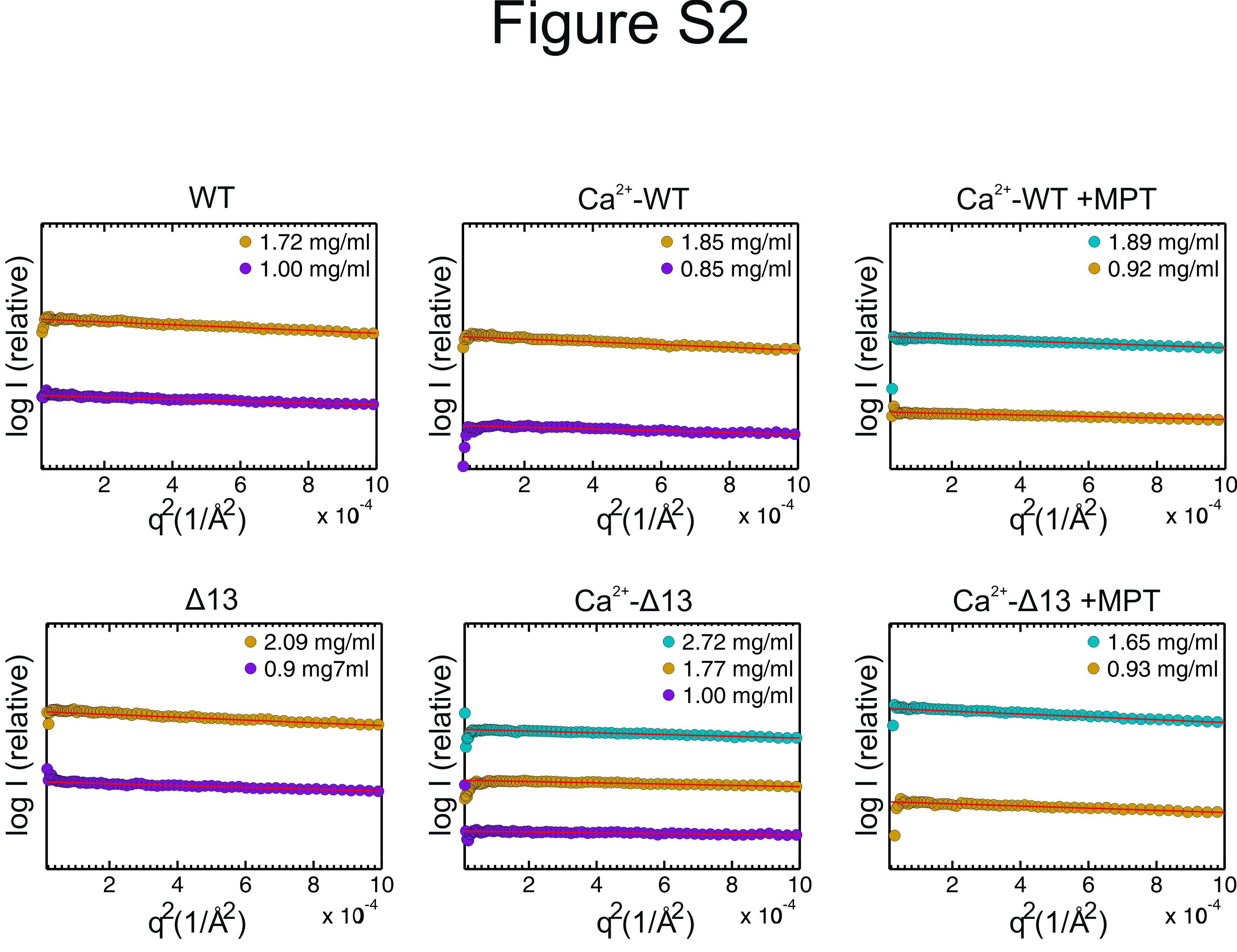

Supplement: Figure S2 — Guinier plots of the datasets that were merged for further data analysis and modeling. The Guinier region was extrapolated to the beam stop (red line). The data sets are plotted in rings with different colors representing different protein concentrations. (TIF) [file pone.0097654.s002.tif]

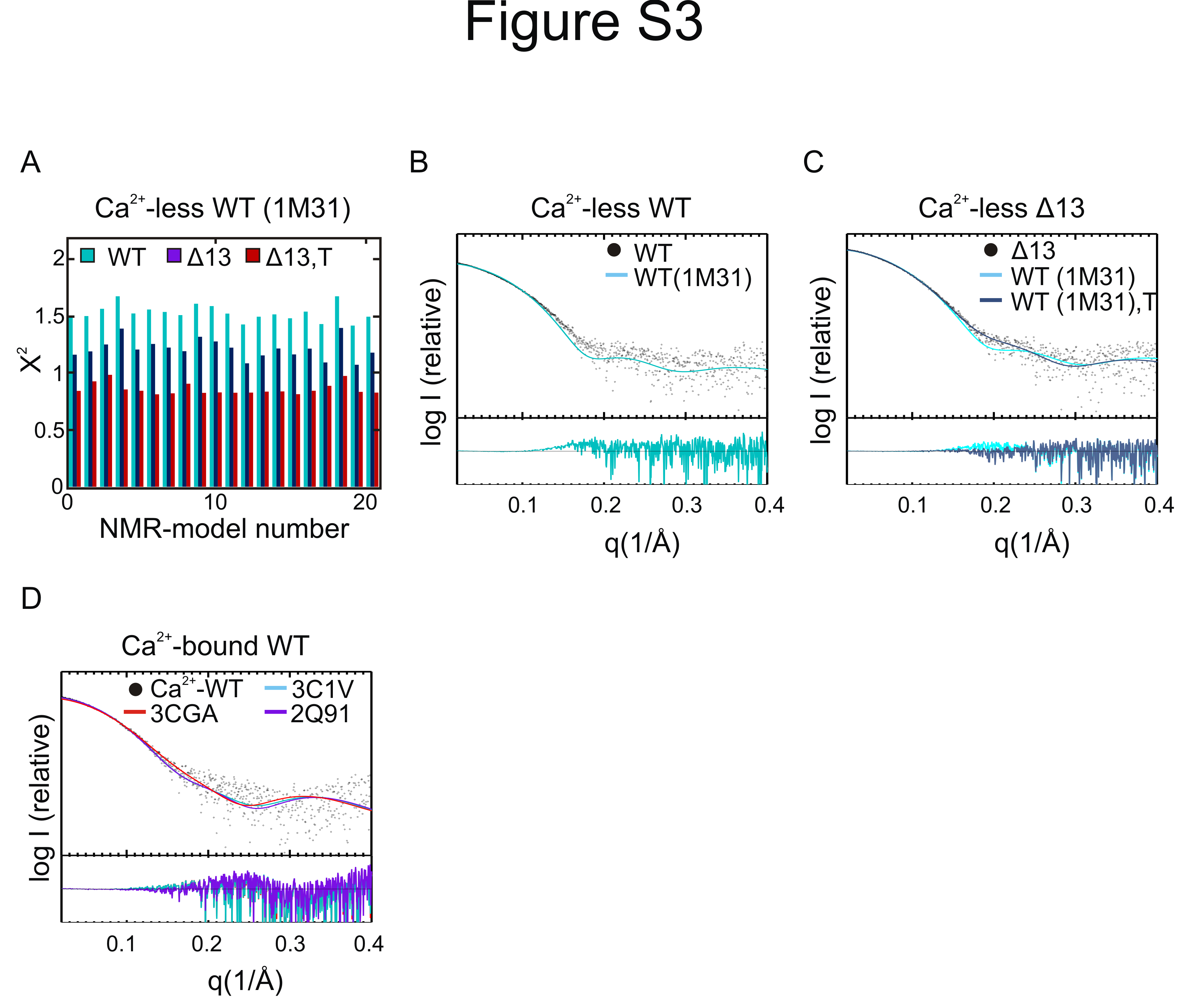

Supplement: Figure S3 — Comparison of the theoretical SAXS intensities of the high-resolution structures with the experimental SAXS scattering. The experimental scattering curves are shown in black circles. The theoretical scattering curves were calculated with the program Crysol (21) and are shown in colored lines. The χ2-values of the individual models (model number 1–20) of the Ca2+-free NMR structure ensemble (PDB code 1M31) [7] were compared to the Ca2+-free SAXS scattering curves (A). The calculated scattering curve of the lowest energy NMR model 1M31 against the Ca2+-WT SAXS (B) and the Ca2+-free Δ13 data (C), respectively. Truncated NMR models are indicated with T. Three Ca2+-bound crystal structures (PDB code 3C1V, 2Q91, 3CGA) are also compared to the Ca2+-bound WT SAXS scattering curve (D). (TIF) [file pone.0097654.s003.tif]

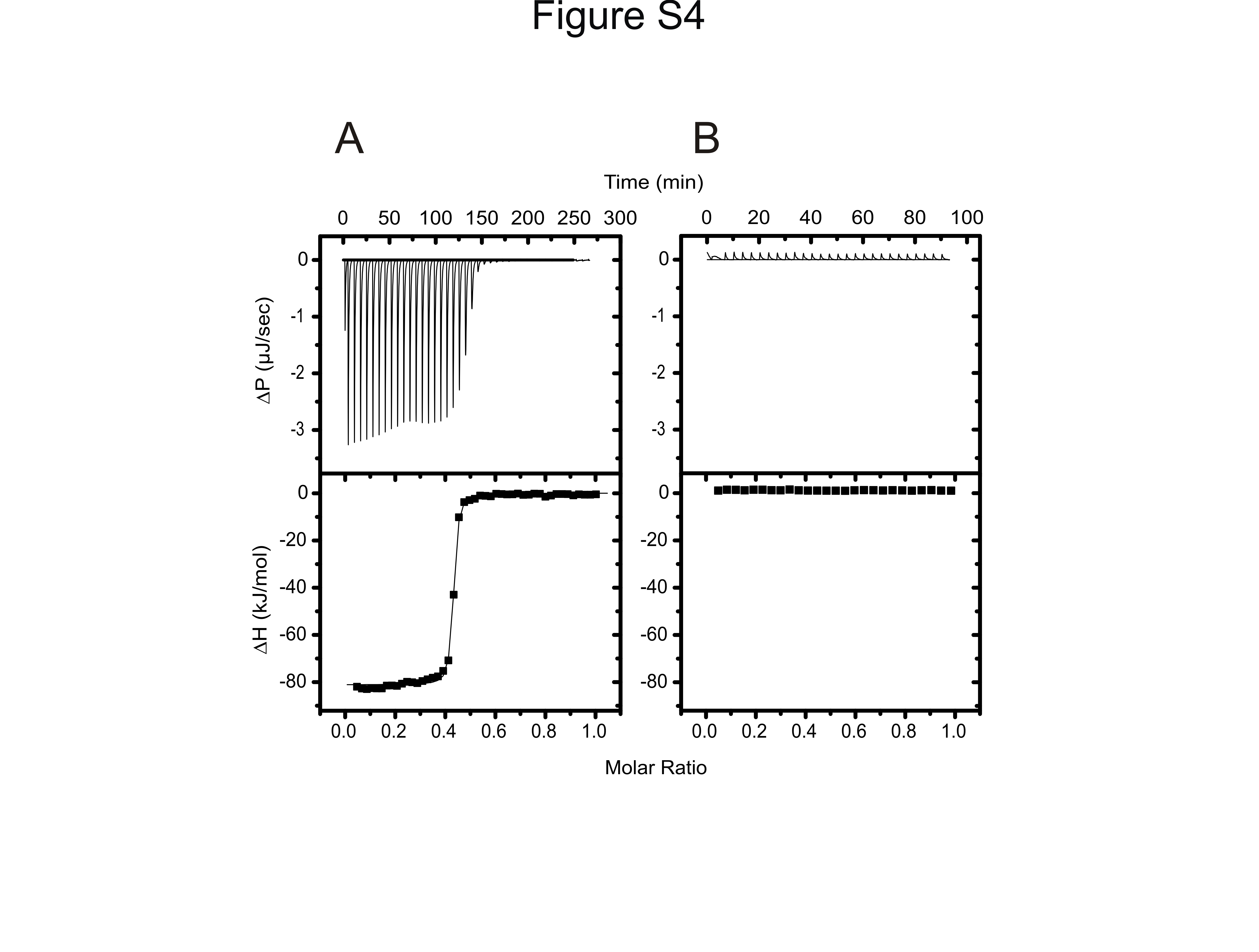

Supplement: Figure S4 — Thermodynamic analysis of S100A4Δ13– MPT interaction. 75 µM S100A4Δ13 titrated with MPT at 25°C in the presence of 1 mM CaCl2 (A) or 1 mM EGTA (B). In the absence of Ca2+ no interaction was detected. Calculated thermodynamic parameters are shown in Table S3. (TIF) [file pone.0097654.s004.tif]

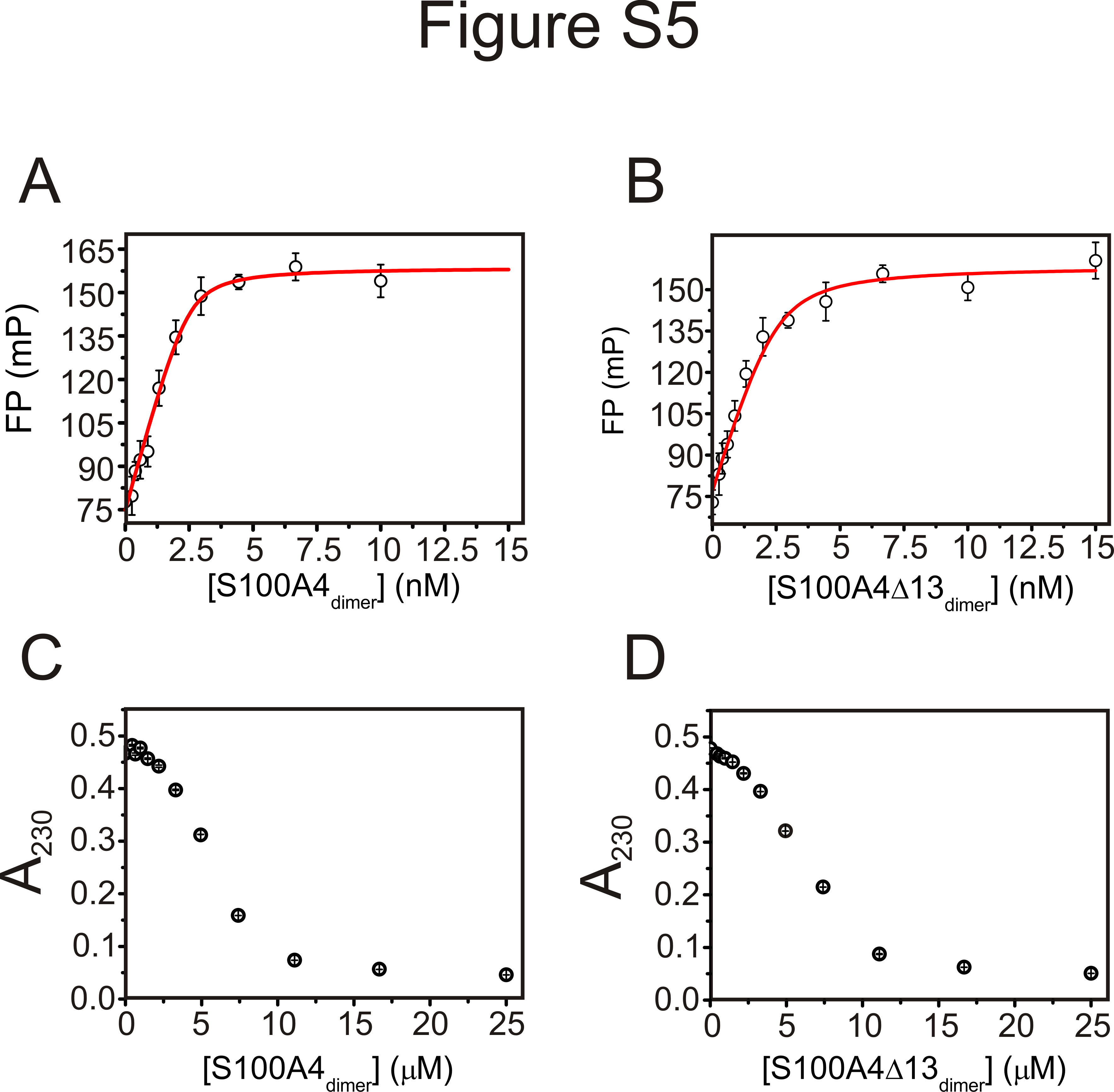

Supplement: Figure S5 — Fluorescence polarization measurements using (A) WT S100A4 and (B) Δ13 S100A4 and fluorescein labelled MPT peptide. Optical density changes at 320 nm when NMIIA 1712Q-1960E rod fragment is titrated with (C) WT and (D) Δ13 S100A4. (TIF) [file pone.0097654.s005.tif]

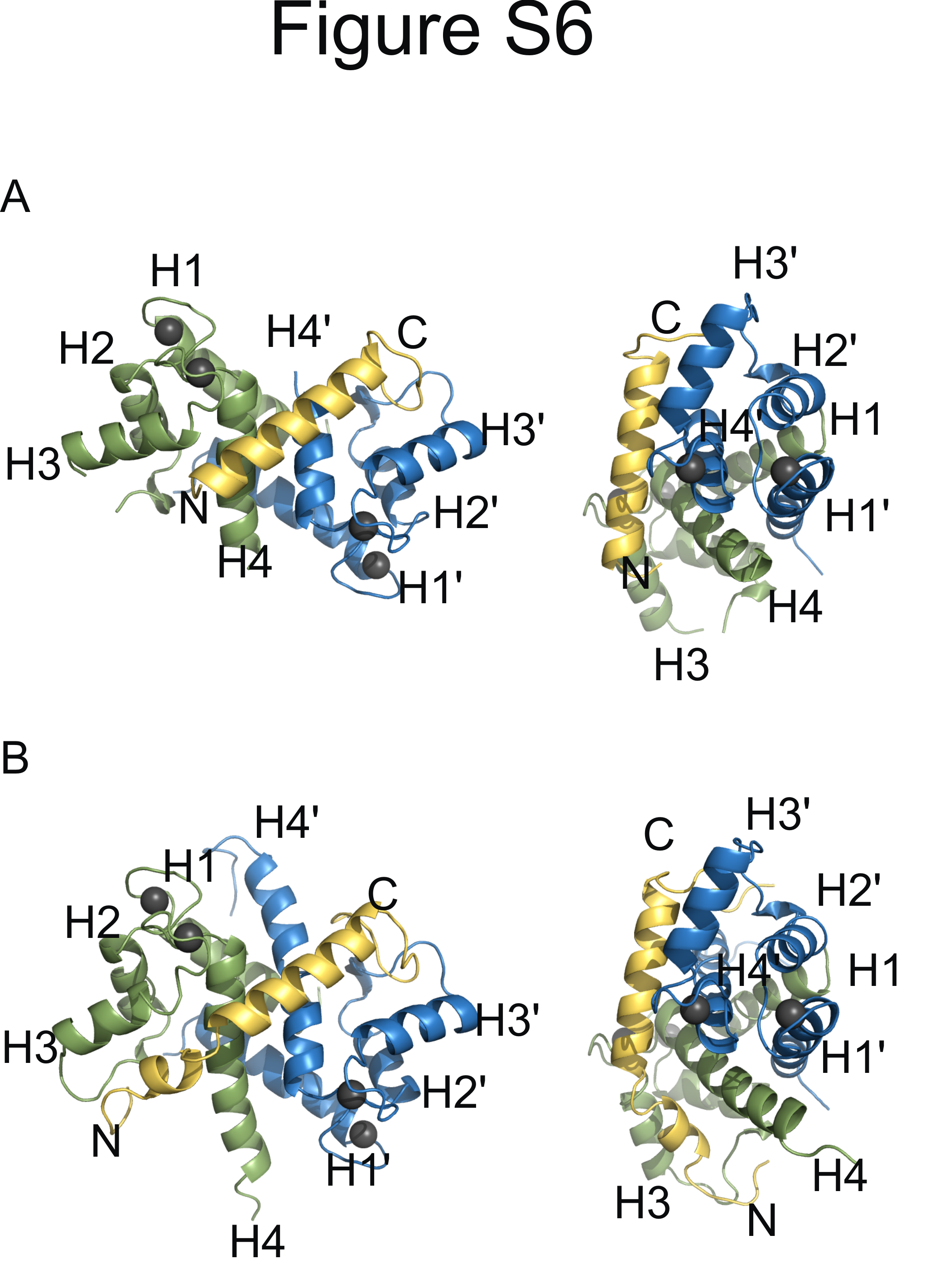

Supplement: Figure S6 — Three-dimensional structure of the Ca2+-activated, MPT-bound Δ13Ser (A) and F45WSer (B) S100A4. Subunit A is shown in green, subunit B in blue and the bound MPT in yellow. Helices (H) and the N- and C-terminus (N, C) of the bound peptide are indicated. (TIF) [file pone.0097654.s006.tif]

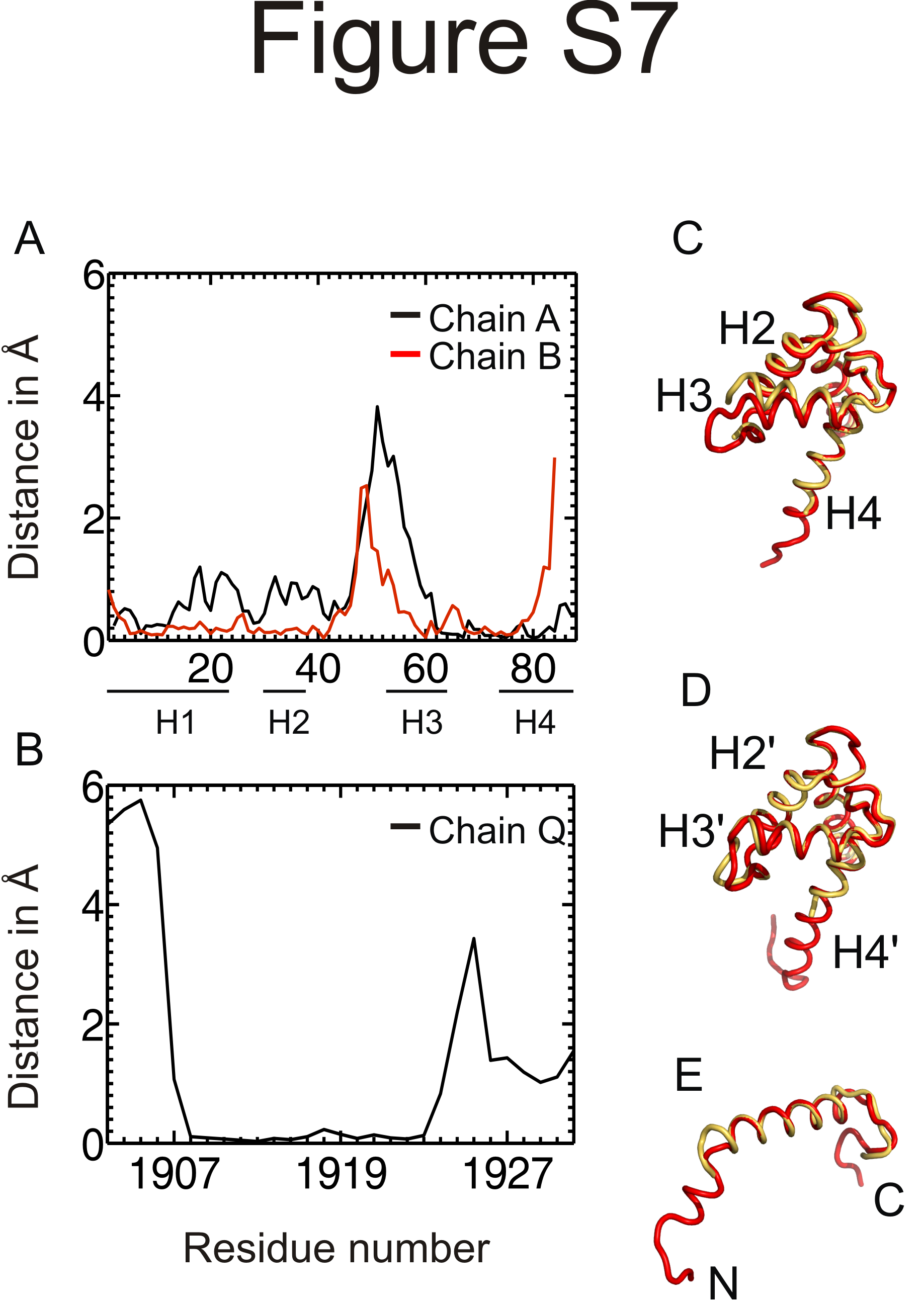

Supplement: Figure S7 — Distance differences in the MPT-bound F45WSer and Δ13Ser. The superposition of the individual chains was performed with LSQMAN [8]. Distance plot of subunit A and B of S100A4 (A) and the bound MPT peptide (B). The superposition of the three dimensional structure of chain A and chain B are shown in (C) and (D), of the bound peptide is shown in (E). (TIF) [file pone.0097654.s007.tif]
