# Supplementary material for: The C-Terminal Random Coil Region Tunes the Ca2+-Binding Affinity of S100A4 through Conformational Activation
Source: PLoS One. 2014 May 15;9(5):e97654. doi: 10.1371/journal.pone.0097654 (PMC4022583; doi:10.1371/journal.pone.0097654)
Supplement: Table S1 — Experimental Rg values and hydrodynamic radius obtained by SAXS and NMR respectively. (DOCX) [file pone.0097654.s008.docx]

**Table S1**

**Table S1:** Experimental R_g_ values and hydrodynamic radius obtained by SAXS and NMR respectively.

| **Sample** | **SAXS concentration**  **(mg/ml)** | **R_g_ (Å)** | **R_h_ (Å)**  **(NMR, 286K)** | **D(m^2^/s)*10^11^** |
| --- | --- | --- | --- | --- |
| WT Ca^2+^-free | 1.0 | 21±1 | 25.6 ± 0.4 | 6.80 ± 0.10 |
| WT Ca^2+^-bound | 0.9 | 22±1 | 33.1 ± 1.6 | 5.27 ± 0.30 |
| ∆13 Ca^2+^-free | 0.9 | 19±1 | 25.8 ± 0.3 | 6.74 ± 0.08 |
| ∆13 Ca^2+^-bound | 1.0 | 19±1 | 24.8 ± 0.6 | 7.03 ± 0.16 |
