# Supplementary material for: The C-Terminal Random Coil Region Tunes the Ca2+-Binding Affinity of S100A4 through Conformational Activation
Source: PLoS One. 2014 May 15;9(5):e97654. doi: 10.1371/journal.pone.0097654 (PMC4022583; doi:10.1371/journal.pone.0097654)
Supplement: Table S2 — Specification of the constructs used in different MD simulations. (DOCX) [file pone.0097654.s009.docx]

**Table S2**

**Table S2:** Specification of the constructs used in different MD simulations.

| ID | Starting structure | Alterations | Ca^2+^ |
| --- | --- | --- | --- |
| WT | 1M31 (1^st^) | None (wild-type) | No |
| WT-Ca^2+^ | 2LNK (1^st^) | Wild-type, NMMIIA peptide removed, | Yes^*^ |
| AAA | 1M31 (1^st^) | R99A, K100A, K101A | No |
| AAA-Ca^2+^ | 2LNK (1^st^) | R99A, K100A, K101A, NMMIIA peptide removed | Yes^*^ |

^*^ Residues ranges 16-28 and 62-73 are removed and replaced by the corresponding regions of structure F45WSer together with the bound Ca^2+^ ions prior energy minimization.
