# Supplementary material for: The C-Terminal Random Coil Region Tunes the Ca2+-Binding Affinity of S100A4 through Conformational Activation
Source: PLoS One. 2014 May 15;9(5):e97654. doi: 10.1371/journal.pone.0097654 (PMC4022583; doi:10.1371/journal.pone.0097654)
Supplement: Table S3 — Thermodynamic parameters of peptide binding of the S100A4 variants determined by ITC measurements. (DOCX) [file pone.0097654.s010.docx]

**Table S3**

| **Table S3:** Thermodynamic parameters of peptide binding of the S100A4 variants determined by ITC measurements* | | | |
| --- | --- | --- | --- |
|  | K_d_ (nM) | ΔH (kJ mol^-1^) | -TΔS (kJ mol^-1^) |
| S100A4 + MPT^a^ | 8.6 ± 1.3 | -63.2 ± 0.2 | 17.2 |
| S100A4Δ13 + MPT | 14.7 ± 1.6 | -81.1 ± 0.2 | 36.3 |
| *Data were obtained as described in Materials and Methods. Note, that the affinity of MPT peptide to S100A4 variants cannot be determined precisely because ITC measurements reach their dynamic range by nanomolar affinities. ^a^Data were obtained previously (2). Standard errors of the fits (SEM) are displayed. | | | |
