# Supplementary material for: The C-Terminal Random Coil Region Tunes the Ca2+-Binding Affinity of S100A4 through Conformational Activation
Source: PLoS One. 2014 May 15;9(5):e97654. doi: 10.1371/journal.pone.0097654 (PMC4022583; doi:10.1371/journal.pone.0097654)
Supplement: Table S4 — Crystallographic table. (DOCX) [file pone.0097654.s011.docx]

**Table S4**

| **Table S4:** Crystallographic table | | |
| --- | --- | --- |
| **Data collection** | **S100A4 Δ13**  (PDB ID: **4CFQ**) | **S100A4 F45WSer** (PDB ID: **4CFR**) |
| Space group | P1 | P4_1_2_1_2 |
| Cell dimensions |  |  |
| a, b, c (Å)  α, β, γ (∘) | 38.7, 38.8, 71.4  88.7 75.5 72.2 | 64.1, 64.1, 138.2 |
| Resolution (Å)^a^ | 69.1-1.4 (1.44-1.37) | 138.2-1.4 (1.48-1.40) |
| *R*_merge_ (%)^b^ | 7.0 (55.2) | 5.9 (76.1) |
| *<I/σ(I)>* | 10.9 (2.2) | 18.6 (2.1) |
| Completeness (%) | 98.5 (94.5) | 99.5 (99.1) |
| Redundancy | 3.5 (2.6) | 9.0 (6.4) |
| **Refinement** |  |  |
| No. observed reflections | 278652 | 519087 |
| No. of unique reflections | 79285 | 57504 |
| *R_work_/ R_free_* (%)^c^ | 16.6/ 19.8 | 16.1/ 17.5 |
| No. atoms |  |  |
| Protein |  |  |
| Chain A and Chain B | 2784 | 3220 |
| Chain C and Chain D | 2775 |  |
| Myosin II fragment, Chain Q | 508 | 717 |
| Myosin II fragment, Chain R | 544 |  |
| Water | 318 | 252 |
| Total | 6929 | 4189 |
| *B*-factors (Å^2^) |  |  |
| Protein |  |  |
| Chain A, Chain B | 22.4, 16.3 | 20.1, 29.9 |
| Chain C, Chain D | 23.9, 17.1 |  |
| Myosin II fragment, Chain Q | 25.5 | 23.4 |
| Myosin II fragment, Chain R | 25.2 |  |
| Water | 28.9 | 34.6 |
| Overall | 21.1 | 25.4 |
| R.m.s. deviations |  |  |
| Bond lengths (Å) | 0.016 | 0.013 |
| Bond angles (°) | 1.343 | 1.152 |
| Ramachandran plot ^d^  Favoured (%) | 96.3 | 98.8 |
| Additional allowed (%) | 2.7 | 1.2 |
| Outlier (%) | 1 | 0 |
| ^a^Values for the highest resolution shell are shown in parentheses  ^b^R_merge_= Ʃ \|*I*_o_ - <*I*>\| / Ʃ \|*I*_o_\| where *I*_o_ observed intensity of a reflection and <*I*> the average intensity from multiple reflections.  ^c^R-factor= Ʃ \|*F*_o_ - *F*_c_\| / Ʃ *F*_o_  ^d^The percentage of peptide bonds in the most favored and additional region of the Ramachandran plot (determined by MolProbity([Chen et al., 2010](#_ENREF_7))) | | |
