# Supplementary material for: The C-Terminal Random Coil Region Tunes the Ca2+-Binding Affinity of S100A4 through Conformational Activation
Source: PLoS One. 2014 May 15;9(5):e97654. doi: 10.1371/journal.pone.0097654 (PMC4022583; doi:10.1371/journal.pone.0097654)
Supplement: Text S1 — Supporting Online Information and Supplementary Methods. (DOCX) [file pone.0097654.s014.docx]

Supporting Online Material

**Crystal structure of the ∆13Ser:MPT and F45WSer:MPT complexes**

The crystal structures of two Ca^2+^-bound S100A4 mutants, the ∆13Ser and F45WSer in complex with MPT (Figure S6) were solved by molecular replacement at the same resolution (1.4 Å) in two different space groups (Table S4). The ∆13Ser mutant displays a similar asymmetric binding mode with the MPT peptide as in the wild-type NMR or the F45WSer complex to MPT and many of the interactions between S100A4 and the MPT are preserved. On the other hand, even though the same MPT peptide was cocrystallized with both mutants, a different length of the MPT was defined in the electron density: in the complex formed with the ∆13Ser mutant a shorter region, residues 1902-1930 is defined compared to residues 1894-1935 in complex with the F45WSer mutant [[1](#_ENREF_1)]. The interaction interface between the ∆13Ser mutants to MPT is approximately 1.3 times reduced with 1802 Å^2^ compared to 2310 Å^2^ for the F45WSer mutant. One reason for this reduction is that part of the interaction interface seen in the F45WSer mutant complex is disordered in the ∆13Ser mutant.

Figure S7 shows the distance plot between the two high-resolution structures revealing differences between each chain. In subunit A the most apparent conformational difference is in helix 3 that normally flanks the N-terminal part of the MPT peptide. In the ∆13Ser structure the position of the helix is more similar to the open, peptide-free configuration. In chain B which holds the C-terminal part of MPT, the differences are slightly less apparent. Helix 3 is also affected albeit to a lesser extent. What may relate to the ∆13 mutation directly is that the conformation of the new C-terminal residues 81-84 deviates more than 1 Å compared to the one observed in chain B of the F45WSer mutant and beyond residue 84 it becomes completely disordered. It appears that the straight helical conformation of helix 4 is more preserved in the F45WSer mutant in the presence of longer C-terminus. In the recent peptide-free Δ8 mutant structure (PDB entry: 4HSZ) similar shortening of the regular α-helix is observed. [[2](#_ENREF_2)]

In the two high-resolution structures the central part of the helical MPT (residues 1908-1918) forms interactions to the core of the S100A4 dimer through helix 3 and 3’, loop 3 and helix 4 and 4’ is the most rigid portion (B-factors 16.3 Å^2^ for Q bound to F45WSer and 13.1 Å^2^ for Q bound to the Δ13Ser). This might be the most crucial interactions between the MPT and S100A4 considering that the MPT is still binding to the ∆13Ser mutant, even if interactions between the N- and the C-terminus of the MPT and the ∆13Ser mutant are strongly reduced, especially in loop 2. By far the largest deviations between the two structures are in the C- and N-terminus of the bound MPT peptide. From residue 1907 and 1921 outwards the conformation rapidly diverges until in the ∆13Ser structure it becomes completely disordered.

In the wild-type NMR structure loop 2 (Phe-A45, Leu-A46, Arg-A49) interactions are formed to the N-terminus of the MPT (Leu-1896, Gln-1897 and Glu-1899). Similar interactions between loop 2 (Trp-A45, Lys-A48, Arg-A49, Asp-A51) and the N-terminus of the MPT (Leu-1896, Gln-1897 and Arg-1898) are observed in the F45WSer mutant. In the ∆13Ser mutant complex this region in the MPT and Lys-A48 and Arg-A49 are not defined. Within the F45WSer mutant complex no interactions between the bound peptide and the deleted part of the C-terminus are observed. The data reported in this paper are tabulated in Table S4 and archived at the PDB database under entries 4CFQ and 4CFR.

Supplementary methods

*Crystallization and structure determination of MPT-bound S100A4*

The complexes were crystallized using the sitting drop vapor diffusion method. The complex solution (1.5 mM) was mixed with precipitant solution (0.2 M ammonium acetate, 0.1 M sodium acetate pH 4.6, 30% w/v PEG 4000 in the case of the F45Wser mutant and 0.1 M MIB buffer pH 4, 25 % w/v PEG 1500 in the case of the Δ13Ser mutant) in an equal amount. Single crystals were mounted and frozen in liquid nitrogen with 10% glycerol as cryoprotectant. X-ray diffraction data were collected on the beamline ID23-1 at the European Synchrotron Radiation Facilities (ESRF, Grenoble, France). Data were collected at 100 K with an X-ray wavelength of 1.073 Å. For the S100A4 Δ13 mutant, four datasets were scaled and merged in XDS [[3](#_ENREF_3)] A molecular replacement solution was found with Phaser [[4](#_ENREF_4)] using the human Ca^2+^-activated, MPT-bound S100A4 quadruple (F45W, C3S, C81S, C86S) mutant (PDB ID code 3zwh) [[1](#_ENREF_1)]. Model building was performed in Coot [[5](#_ENREF_5)] and refinement in Phenix [[6](#_ENREF_6)]. The model was refined using anisotropic atomic displacement parameters and riding hydrogens were added. ∆13Ser mutant crystallized readily in the presence of MPT, but the space group of the crystal changed in the absence of the C-terminal interactions compared to the F45WSer mutant complex. As a consequence one MPT-bound to one homodimer was found in the asymmetric unit of the crystals of the F45WSer mutant (chains A, B, Q), whereas for the ∆13Ser mutant two MPT-bound homodimers were found per asymmetric unit (chains A, B, Q and C, D, R). Between the two complexes in the asymmetric unit there are only subtle differences, therefore we mostly focus on chain A, B and Q in the analysis.

*Fluorescence polarization measurement*

The NMIIA fragment 1894R-1937K with a N-terminal cysteine was expressed and purified as the MPT fragment. The RP-HPLC purified peptide was dissolved in 100 mM Hepes pH 7.5, supplemented with 1 mM TCEP, and a 5-fold molar excess of 5-iodoacteamidofluorescein (Sigma-Aldrich #I-9271) was added to the peptide solution. After 2 hours of incubation at room temperature in the dark the fluorescein-conjugated MPT (Fluo-MPT) was separated by RP-HPLC. The lyophilized peptide was dissolved in DMSO and stored at -20°C prior to use.

In order to determine the affinity of wild-type and deletion mutant S100A4 2.5 nM Fluo-MPT was diluted in FP assay buffer (20 mM Hepes pH 7.5, 150 mM NaCl, 1 mM CaCl_2_, 0.1 mM TCEP, and 0.05% Tween-20) with different S100A4 concentrations (15 – 0.26 nM). After 2 hours of incubation at room temperature the fluorescence polarization of each data point was measured in triplicates in a 384-well microplate (Corning #3676) on the Synergy H4 multi-mode microplate reader (BioTek). The data was fitted to:

$$FP=FP_{\min}+(FP_{\max}-FP_{\min})\frac{K_{d}+\left[S\right]+\left[M\right]-\sqrt{(K_{d}+\left[S\right]+\left[M\right])^{2}-4\left[S\right]\left[M\right]}}{2\left[M\right]}$$

where K_d_ is the dissociation constant, [S] the total concentration of S100A4, [M] the total concentration of fluorescein labeled MPT, FP the measured fluorescence polarization of the reaction mixture, and FP_min_ and FP_max_ the fluorescence polarization values of the solution when the total amount of Fluo-MPT is free or complexed, respectively.

*Filament disassembly assay*

5 µM NMIIA 1712Q-1960E rod was titrated with S100A4 variants in triplicates in buffer containing 20 mM Hepes pH 7.5, 100 mM NaCl, 1 mM CaCl_2_, 2 mM MgCl_2_, 0.1 mM TCEP. The absorbance at 320 nm of each data point (350 µL) was measured in a 96-well microplate (Nunc #269620) on the Synergy H4 multi-mode microplate reader (BioTek).

**References**

1. Kiss B, Duelli A, Radnai L, Kekesi KA, Katona G, et al. (2012) Crystal structure of the S100A4-nonmuscle myosin IIA tail fragment complex reveals an asymmetric target binding mechanism. Proc Natl Acad Sci U S A 109: 6048-6053.

2. Ramagopal UA, Dulyaninova NG, Varney KM, Wilder PT, Nallamsetty S, et al. (2013) Structure of the S100A4/myosin-IIA complex. BMC Struct Biol 13: 31.

3. Kabsch W (2010) Xds. Acta Crystallogr D Biol Crystallogr 66: 125-132.

4. McCoy AJ (2007) Solving structures of protein complexes by molecular replacement with Phaser. Acta Crystallogr D Biol Crystallogr 63: 32-41.

5. Emsley P, Cowtan K (2004) Coot: model-building tools for molecular graphics. Acta Crystallographica Section D-Biological Crystallography 60: 2126-2132.

6. Adams PD, Afonine PV, Bunkoczi G, Chen VB, Davis IW, et al. (2010) PHENIX: a comprehensive Python-based system for macromolecular structure solution. Acta Crystallogr D Biol Crystallogr 66: 213-221.

7. Vallely KM, Rustandi RR, Ellis KC, Varlamova O, Bresnick AR, et al. (2002) Solution structure of human Mts1 (S100A4) as determined by NMR spectroscopy. Biochemistry 41: 12670-12680.

8. Kleywegt GJ (1999) Experimental assessment of differences between related protein crystal structures. Acta Crystallogr D Biol Crystallogr 55: 1878-1884.

**Figure legends**

**Figure S1** Typical ^1^H protein signal decay curve as function of the applied gradient strength. Points indicate measured values, while the continuous line is the fitted curve.

**Figure S2** Guinier plots of the datasets that were merged for further data analysis and modeling. The Guinier region was extrapolated to the beam stop (*red line*). The data sets are plotted in rings with different colors representing different protein concentrations.

**Figure S3** Comparison of the theoretical SAXS intensities of the high-resolution structures with the experimental SAXS scattering. The experimental scattering curves are shown in black circles. The theoretical scattering curves were calculated with the program Crysol (21) and are shown in colored lines. The χ^2^-values of the individual models (model number 1-20) of the Ca^2+^-free NMR structure ensemble (PDB code 1M31) [[7](#_ENREF_7)] were compared to the Ca^2+^-free SAXS scattering curves (A). The calculated scattering curve of the lowest energy NMR model 1M31 against the Ca^2+^-WT SAXS (B) and the Ca^2+^-free ∆13 data (C), respectively. Truncated NMR models are indicated with T. Three Ca^2+^-bound crystal structures (PDB code 3C1V, 2Q91, 3CGA) are also compared to the Ca^2+^-bound WT SAXS scattering curve (D).

**Figure S4** Thermodynamic analysis of S100A4Δ13 – MPT interaction. 75 µM S100A4Δ13 titrated with MPT at 25°C in the presence of 1 mM CaCl_2_ (A) or 1 mM EGTA (B). In the absence of Ca^2+^ no interaction was detected. Calculated thermodynamic parameters are shown in Table S3.

**Figure S5** Fluorescence polarization measurements using (A) WT S100A4 and (B) ∆13 S100A4 and fluorescein labelled MPT peptide. Optical density changes at 320 nm when NMIIA 1712Q-1960E rod fragment is titrated with (C) WT and (D) ∆13 S100A4.

**Figure S6** Three-dimensional structure of the Ca^2+^-activated, MPT-bound ∆13Ser (A) and F45WSer (B) S100A4. Subunit A is shown in *green*, subunit B in *blue* and the bound MPT in *yellow*. Helices (H) and the N- and C-terminus (N, C) of the bound peptide are indicated.

**Figure S7** Distance differences in the MPT-bound F45WSer and ∆13Ser. The superposition of the individual chains was performed with LSQMAN [[8](#_ENREF_8)]. Distance plot of subunit A and B of S100A4 (A) and the bound MPT peptide (B). The superposition of the three dimensional structure of chain A and chain B are shown in (C) and (D), of the bound peptide is shown in (E).

**Movie S1** MD trajectories of the Ca^2+^-free S100A4. The time interval visualized is between 20 ns and 100 ns. The last 17 residues of the C-terminal region are shown in red and the EF-hand motifs in cyan.

**Movie S2** MD trajectories of the Ca^2+^-bound S100A4. The time interval visualized is between 20 ns and 100 ns. The last 17 residues of the C-terminal region are shown in red and the EF-hand motifs in cyan.

**Table S1** Experimental R_g_ values and hydrodynamic radius obtained by SAXS and NMR respectively.

**Table S2** Specification of the constructs used in different MD simulations.

**Table S3** Thermodynamic parameters of peptide binding of the S100A4 variants determined by ITC measurements.

**Table S4** Crystallographic table
